# Regulation of the Epithelial-Mesenchymal Transition by Claudin-3 and Claudin-4

**DOI:** 10.1371/journal.pone.0067496

**Published:** 2013-06-21

**Authors:** Xinjian Lin, Xiying Shang, Gerald Manorek, Stephen B. Howell

**Affiliations:** Department of Medicine and the Moores UCSD Cancer Center, University of California San Diego, La Jolla, California, United States of America; Aix-Marseille University, France

## Abstract

The mechanisms that control intracellular adhesion are central to the process of invasion and metastasis. Claudin-3 (CLDN3) and claudin-4 (CLDN4) are major structural molecules of the tight junctions that link epithelial cells. Our prior work has demonstrated that knockdown of the expression of either CLDN3 or CLDN4 produces marked changes in the phenotype of ovarian carcinoma cells including increases in growth rate *in vivo*, migration, invasion, metastasis, and drug resistance, similar to those produced by the epithelial-to-mesenchymal transition (EMT). We postulated that these changes may result from the ability of CLDN3 or CLDN4 to suppress EMT. In this study we found that knockdown of either CLDN3 or CLDN4 increased cell size and resulted in flattened morphology. While knockdown of CLDN3 or CLDN4 did not alter the expression of vimentin, it significantly down-regulated the level of E-cadherin and up-regulated N-cadherin expression. Conversely, over-expression of CLDN3 or CLDN4 in a cell line that does not express endogenous CLDN3 or CLDN4 decreased N-cadherin expression. Re-expression of E-cadherin in the CLDN3 or CLDN4 knockdown cells reduced migration, invasion and tumor growth *in vivo*. Loss of either CLDN3 or CLDN4 resulted in activation of the PI3K pathway as evidenced by increased Akt phosphorylation, elevated cellular PIP3 content and PI3K activity as well as up-regulation of the mRNA and protein levels of the transcription factor Twist. Taken together, these findings suggest that CLDN3 and CLDN4 function to sustain an epithelial phenotype and that their loss promotes EMT.

## Introduction

Epithelial cells adhere tightly to their neighbors through specialized junctional complexes including tight junctions. Cell–cell adhesion is important in maintaining epithelial morphology and is closely linked to cell proliferation and migration [1]. During embryogenesis, epithelial cells can undergo an epithelial–mesenchymal transition (EMT) which endows them with mesenchymal characteristics and facilitates migration through the extracellular matrix (ECM) and settlement in new areas for subsequent organogenesis. The wound healing process is another form of physiological EMT whereas tissue fibrosis and cancer progression are examples of pathological EMT [2], [3], [4]. In the context of cancer, it is generally accepted that malignant cells arising in epithelia lose their epithelial characteristics and acquire certain mesenchymal properties that promote ECM invasion and distant metastasis in an EMT-like process. Epithelial ovarian cancer is unique in its metastatic pattern. Emanating from an intra-abdominal organ, the neoplastic ovarian epithelial cells must detach from the primary ovarian tumor, resist anoikis, and negotiate attachment and invasion to form a metastatic site. To attach to the submesothelial ECM, the cells must rearrange intercellular adhesions and then invade through the mesothelial cell layer and into the ECM [5]. The loss of tight junctions (TJ) in carcinomas is regarded as an important step in the process that leads to loss of cell–cell adhesion and metastasis [6], [7].

The claudin family consists of 27 members that exhibit distinct developmental stage and tissue-specific expression [8], [9]. Claudin expression is deregulated in cancer and varies across different epithelial tumor types. “Claudin-low” breast cancer is associated with poor survival [10], [11], [12] and this is also true for pancreatic [13] and colon cancer [14], [15]. Several members of claudin family including CLDN3, 4, 7 and 10 have been reported to be more highly expressed in ovarian carcinoma compared to normal ovarian surface epithelium [16], [17], [18], [19], [20]. However, studies on the prognostic significance of these claudins in ovarian carcinoma have yielded conflicting results. Low CLDN3 protein expression was associated with a trend toward poorer survival in an analysis of 115 primary ovarian carcinomas [21]. In contrast, in another study high CLDN3 expression in ovarian serous adenocarcinoma was correlated with shorter survival in both univariate and multivariate analyses [22]. Thus, definite correlation between expression and clinical significance of the claudin proteins in ovarian carcinoma remains controversial. In addition, although more than 70% of high grade serous carcinomas express abundant amounts of CLDN3 and CLDN4, the site of origin of such tumors also remains in some doubt. Since the normal ovarian surface epithelium does not express either of these proteins, if serous carcinomas arise from this epithelium then a fraction must undergo a mesenchymal-to-epithelial transition (MET) during which the expression of CLDN3 and CLDN4 is up-regulated. However, the distal Fallopian tube is now thought to be the source of many high-grade serous ovarian cancers [23], [24], [25], [26], and we have reported that this epithelium expresses high levels of both proteins [27]. Thus, those tumors arising from this site may already be endowed with the ability to express these claudins in abundance.

Like the adherens junction, the TJ complex appears to be an important source of signals that restrain growth and metastatic potential in ovarian cancers. Studies in experimental models using human cell lines suggest that those tumors in which the up-regulation of CLDN3 or CLDN4 expression is incomplete, or in which expression is subsequently lost, TJ function and signaling is compromised resulting in enhanced growth rate, migration, invasion, metastatic potential and drug resistance [27], [28]. These results lead to the prediction that those ovarian carcinomas that express only low levels of CLDN3 and CLDN4 will have greater aggressiveness and metastatic propensity, and that disabling TJ function in those that express high levels of these proteins will launch an EMT and result in a more mesenchymal phenotype.

We hypothesized that loss of CLDN3 or CLDN4 activates an EMT in ovarian cancer cells. Consistent with this our previous work demonstrated that knockdown of either CLDN3 or CLDN4 produced marked changes in the phenotype of putative human ovarian cancer cells that include increased growth rate *in vivo*, enhanced cell motility and metastatic potential as well as resistance to platinum-containing drugs, all of which are EMT-related characteristics [29]. In the present study we examined the effect of knocking down the expression of either CLDN3 or CLDN4 on known markers of EMT and report here that CLDN3 and CLDN4 suppression promotes EMT in ovarian cancer cells as reflected by a marked decrease in E-cadherin, up-regulation of Twist and activation of the PI3K/AKT pathway.

## Materials and Methods

### Cells, cell culture and transfection

The human carcinoma cell lines 2008 and HEY were grown in RPMI 1640 supplemented with 10% fetal bovine serum (FBS), 100 U/ml penicillin, 100 mg/ml streptomycin. Both of these cell lines were reported to be isolated from patients with ovarian cancer [30], [31]. CLDN3- or CLDN4-expressing sublines of HEY (HEY-CLDN3 and HEY-CLDN4, respectively) and empty vector-transfected control (HEY-EV) [27] were grown in the same complete medium as 2008 and HEY cells. The 2008 CLDN3 or CLDN4 knockdown sublines (CLDN3KD and CLDN4KD, respectively) and scrambled shRNAi control cell line (2008-scb) [27], [28], [32] were cultured in the presence of 10 µg/ml puromycin. Cultures were maintained at 37°C in a humidified atmosphere of 5% CO_2_ and 95% air. To generate 2008-Ecad, CLDN3KD-Ecad and CLDN4KD-Ecad lines that stably expressed GFP-E-cadherin, we transfected 2008, CLDN3KD and CLDN4KD cells with pcDNA3.1-E-cadherin-GFP construct [33] or empty vector control pcDNA3.1(+) (Invitrogen, Carlsbad, CA) using the FuGene6 reagent (Roche Diagnostics) according to the manufacturer's instructions. Stable transfectants were established after 2 weeks of selection in medium with 400 µg/ml geneticin.

### Cloning and lentivirus transduction

To express CLDN3 or CLDN4 in the HEY human ovarian cell line in which CLDN3 and CLDN4 are undetectable [34], the full-length human CLDN3 or CLDN4 cDNA originally in a pCIneo vector [34] was subcloned into pLVX-mCherry vector using In-Fusion cloning strategy (Clontech). The resulting vectors were sequence-verified. For lentivirus production, LentiX-293T cells (Clontech) were transfected with the CLDN3- or CLDN4-expressing vector and Lenti-X HTX Packaging Mix containing lentiviral packaging genes using the Xfect transfection reagent according to the manufacturer’s protocol (Clontech). The produced lentiviruses were used to transduce the HEY cells separately and a pool of cells expressing high levels of the fluorescent mCherry tag were isolated by FACS sorting and designated as HEY-CLDN3 and HEY-CLDN4. HEY cells infected with lentiviruses containing mCherry only were used as control (HEY-EV).

### Western blot analysis

Cells grown to 80% confluence were removed from the dishes with Cell Dissociation Buffer (enzyme free, PBS-based; Invitrogen, catalog number 13151-014), washed twice with Dulbecco's phosphate buffered saline. Whole-cell lysates were prepared in RIPA lysis buffer (mammalian cell lysis kit, MCL-1; Sigma-Aldrich) with protease inhibitors and centrifuged at 14,000 × g for 20 min at 4 °C. The protein was loaded on SDS-PAGE and separated by electrophoresis. A Bio-Rad Trans-Blot system was used to transfer the proteins to Immobilon-P FL membranes (Millipore). Membranes were blocked for 1 h at room temperature in the Odyssey Blocking Buffer (Li-Cor Biosciences), followed by incubation overnight at 4°C with specific antibodies. The following primary antibodies were used: rabbit polyclonal anti-claudin 3 (Invitrogen), mouse monoclonal anti-claudin 4 (Abcam), rabbit monoclonal anti-E-cadherin, anti-N-cadherin, anti-Vimentin, anti-Snail, anti-Slug, anti-Zeb1 (Epithelial-Mesenchymal Transition Antibody Sampler Kit #9782, Cell Signaling Technology), mouse monoclonal anti-Zeb2 (Novus Biologicals), mouse monoclonal anti-Twist (Santa Cruz Biotechnology), rabbit monoclonal anti-phospho-Akt (Cell Signaling Technology), rabbit monoclonal anti-Akt (pan) (Cell Signaling Technology), mouse monoclonal anti-α-tubulin (Sigma-Aldrich), mouse monoclonal or rabbit polyclonal anti-β-actin antibody (Santa Cruz Biotechnology). After washing 4 times for 5 min each at room temperature in PBS containing 0.1% Tween 20 the blots were incubated for 1 h at room temperature with fluorescently labeled secondary antibody (Li-Cor Biosciences) diluted 1∶10,000 in the Odyssey Blocking Buffer containing 0.1% Tween 20 and 0.02% SDS. After 4 washes for 5 min each in 0.1% Tween 20 PBS and rinse with PBS the blots probed with fluorescently labeled antibody were imaged using an Odyssey Infrared Imager (Li-Cor Biosciences).

### Cell spreading and morphology

The 2008, CLDN3KD and CLDN4KD cells were plated onto 6-well cell culture plates at 2×10^5^ cells/well and incubated at 37°C overnight. Cells were then fixed and stained for F-actin with Alexa Fluor 488-labeled phalloidin (Invitrogen). The nuclei were stained with 0.1% Hoechst 33342 (Sigma-Aldrich). Images were acquired by a Nikon inverted microscope with a 20 × dry objective and photographed with a Hamamatsu Cooled CCD camera. Cell areas were determined using MetaMorph software. Cells were manually outlined based on phalloidin labeling of actin, and the area was calculated in µm^2^ using an automation tool in MetaMorph software. Only cells not in contact with neighboring cells were analyzed. Super-resolution 3D images of cells stained for F-actin were acquired with a DeltaVision OMX imaging system (Applied Precision).

### Phosphatidylinositol-3 kinase (PI3K) and phosphatidylinositol (3,4,5)-triphosphate (PIP3) assays

PI3K activity assay was performed with an enzyme-linked immunosorbent assay (ELISA) K-1000s-PI3-kinase activity (ELISA:Pico, Echelon Biosciences Inc., Salt Lake City, UT,) according the manufacturer's instructions. The amount of PIP3 was measured with the competitive PIP3 mass ELISA kit (K-2500s, Echelon Biosciences, Inc.) as per the manufacturer’s instructions.

### Cell migration using wound-healing/scratch assay

Cells were grown to confluence on 6-well cell culture plates and a same size scratch was made through the cell monolayer using a pipette tip. After washing with HBSS, fresh culture medium was added and the cells were incubated at 37°C in a humid environment with 5% CO_2_. Wound closure was photographed every 15 minutes by the Nikon Eclipse Ti-S/L 100 microscope system. The experiment was performed twice and assayed in triplicate.

### Transwell invasion assay

The invasion capabilities of the cells were determined using a modified Boyden chamber invasion assay. Cells were cultured to about 80% confluence and serum starved overnight. Transwell inserts (BD Biosciences) of 8-µm pore size were coated with 150 µL of 1∶60 diluted Matrigel (BD Biosciences). 300,000 cells after overnight starvation were plated onto the top of each of the coated filters in 150 µL serum-free medium. 300 µL of the same medium containing 20% FCS was placed in the lower chamber (i.e., the well beneath the filter) to act as a chemoattractant. After 24 hr of culture, cells that did not migrate or invade through the pores of the Transwell inserts will be manually removed with cotton swabs and the inserts were fixed in cold methanol for 10 min and then stained with 0.01% crystal violet in 20% ethanol. After washing thoroughly colorimetric readings were taken at 595 nm. The experiment was performed twice with each sample in triplicate.

### *In vivo* experiments

All work performed with animals was in accordance with and approved by the Institutional Animal Care and Use Committee (IACUC) at the University of California, San Diego. Ten female BALB/c-nu/nu mice, 7–8 weeks old (Charles River Laboratories, Wilmington, MA), were inoculated SC with 1×10^6^ 2008-EV, 2008-Ecad, CLDN3KD and CLDN3KD-Ecad, CLDN4KD and CLDN4KD-Ecad bilaterally into the left and right axillary and flank region. After the tumors were palpable, tumor volume was determined every two days by the formula: volume  =  length × width^2^/2 and plotted as a function of time to generate the *in vivo* growth curves.

### Statistical analysis

All two-group comparisons utilized Student’s *t*-test with the assumption of unequal variance. Data are presented as mean ± SEM determined from a minimum of 3 independent experiments.

## Results

### Effect of CLDN3 and CLDN4 knockdown on cell spreading and morphology

**Figure 1:**
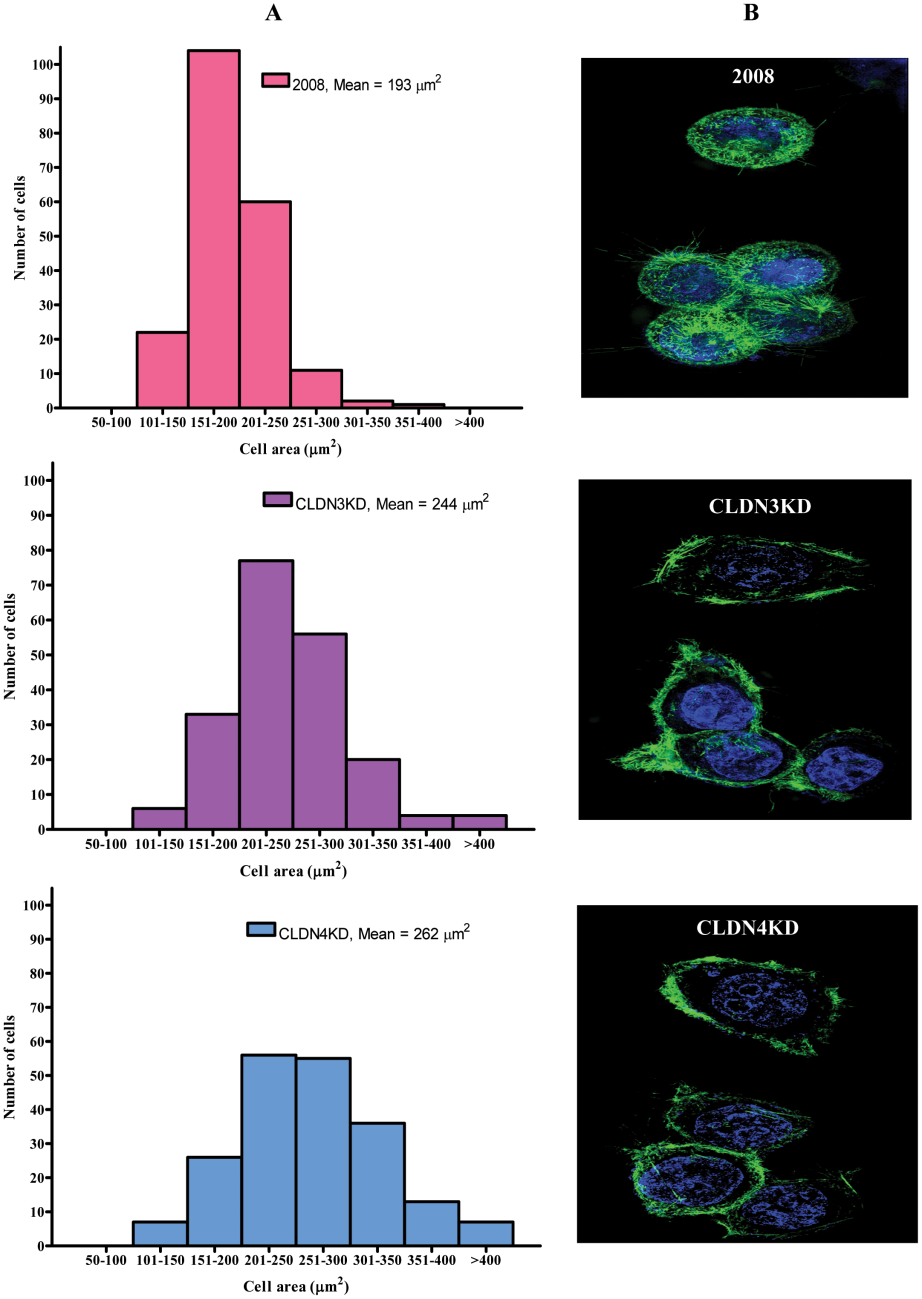
Effect of CLDN3 and CLDN4 knockdown on cell spreading and morphology. (A) Size distribution of 2008, CLDN3KD and CLDN4KD cells at 16 h after seeding as determined by image analysis software. (B) Super-resolution images of the 2008, CLDN3KD and CLDN4KD cells stained with Alexa Fluor 488 Phalloidin (green) demonstrating redistribution of F-actin fibers. The nuclei were stained with 0.1% Hoechst 33342 (blue).

We have previously prepared sublines of the human epithelial cell line 2008, designated as CLDN3KD and CLDN4KD, in which CLDN3 or CLDN4 was constitutively knocked down by infection with a lentivirus expressing an shRNAi targeting to one or the other of these two claudins [27]. In the CLDN3KD cells, the CLDN3 mRNA level was reduced to 5% of control and the protein level to 26% of control. In the CLDN4KD cells, the CLDN4 mRNA level was reduced to 12% of control and the protein level to 16% of control. Transduction of 2008 cells with a vector expressing a scrambled shRNAi that did not target any human gene did not alter the mRNA or protein level of either CLDN3 or CLDN4 [27]. We showed that knockdown of either CLDN3 or CLDN4 substantially increased tumor growth and metastases [27], which prompted us to postulate that these two claudins may function to suppress EMT. Since cell spreading is an important aspect of adhesion, and changes in adhesion characteristically accompanies EMT, we first tested the influence of CLDN3 and CLDN4 on cell spreading. Cell area was quantitatively determined 16 h after plating the cells on tissue culture dishes. As shown in Figure 1A, parental 2008 cells exhibited an average projected area of 193 µm^2^ whereas the CLDN3KD and CLDN4KD cells had mean areas of 244 (p = 0.034) and 262 µm^2^ (p = 0.006). Notably, 93% of the parental 2008 cells had cell areas <250 µm^2^ whereas only 58% and 44.5% of the CLDN3KD and CLDN4KD cells, respectively, were this small. Thus, knockdown of CLDN3 or CLDN4 caused significantly more cell spreading. In addition, as shown in Figure 1B, the morphology and pattern of distribution of F-actin fibrils was altered when the claudins were knocked down. Once attached, the majority of the parental 2008 cells remained rounded and the F-actin was distributed in the area between cytoplasmic and nuclear membrane. In contrast, the CLDN3KD and CLDN4KD cells spread considerably more with extended and flattened morphology and the majority of F-actin fibrils were observed in the periphery of the cell.

### CLDN3 and CLDN4 modulate expression of EMT markers

**Figure 2:**
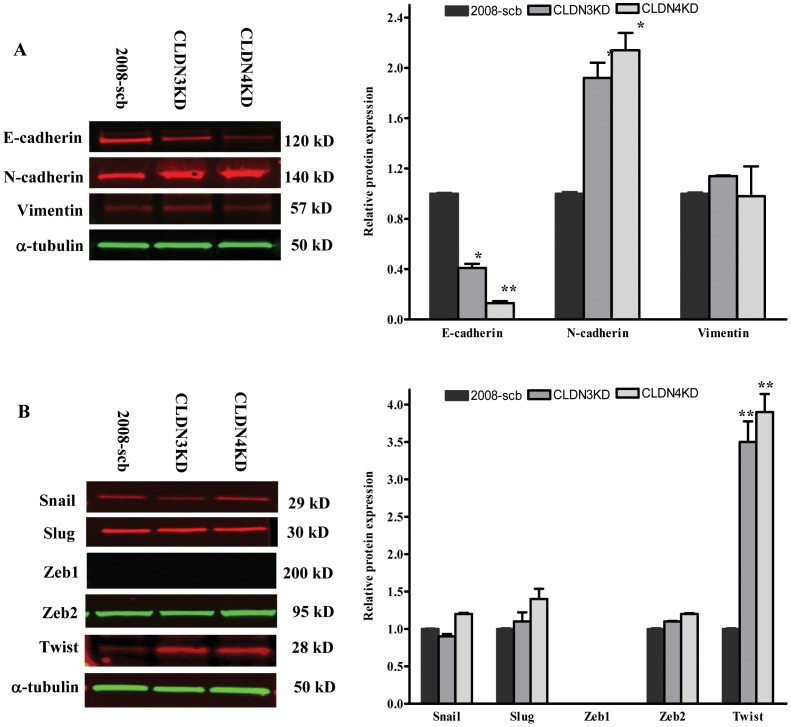
CLDN3 and CLDN4 regulate the expression of EMT markers. (A) Western blot analysis of the expression of E-cadherin and mesenchymal markers N-cadherin and vimentin in the scrambled control 2008 (2008-scb), CLDN3KD and CLDN4KD cells. (B) Western blot analysis of the levels of EMT-inducing transcription factors. The histograms indicate the levels of the protein determined from 3 independent experiments expressed as the mean ratio relative to that in the 2008-scb cells after normalization to α-tubulin. Values are mean ± SEM. *p<0.05 *versus* 2008-scb; **p<0.01 *versus* 2008-scb.

The increased cell spreading and acquisition of an extended and flattened morphology suggested that knockdown of CLDN3 or CLDN4 initiated an EMT. To explore this, the expression of markers associated with EMT was quantified in the parental and knockdown cells. As shown in Figure 2A, knockdown of CLDN3 or CLDN4 reduced the level of E-cadherin to 41±5.6% (p = 0.024) and 13±2.7% (p = 0.007) respectively, of that in the 2008-scb cells. In contrast, there was 1.9±0.21-fold (p = 0.038) and 2.1±0.24-fold (p = 0.032) increase, respectively, in the expression of N-cadherin in the CLDN3KD and CLDN4KD cells. No significant changes in the expression levels of vimentin were detected.

Given that CLDN3 and CLDN4 knockdown reduced E-cadherin, and the known correlation between loss of E-cadherin expression and activation of EMT [35], [36], we asked whether knockdown of CLDN3 or CLDN4 increased the expression of transcription factors known to drive EMT including Snail, Slug, Zeb1, Zeb2 and Twist. As shown in Figure 2B, Western blot analysis revealed that neither Snail, Slug nor Zeb2 expression was affected by CLDN3 or CLDN4 status, and Zeb1 was not expressed at all in 2008, CLDN3KD or CLDN4KD cells. In contrast, Twist expression was significantly enhanced by 3.5±0.48-fold (p = 0.005) in the CLDN3KD cells, and by 3.9±0.42-fold (p = 0.002) in the CLDN4KD cells, compared with the 2008-scb cells. These data support the notion that CLDN3 and CLDN4 promote cell migration and invasion via activation of an EMT.

**Figure 3:**
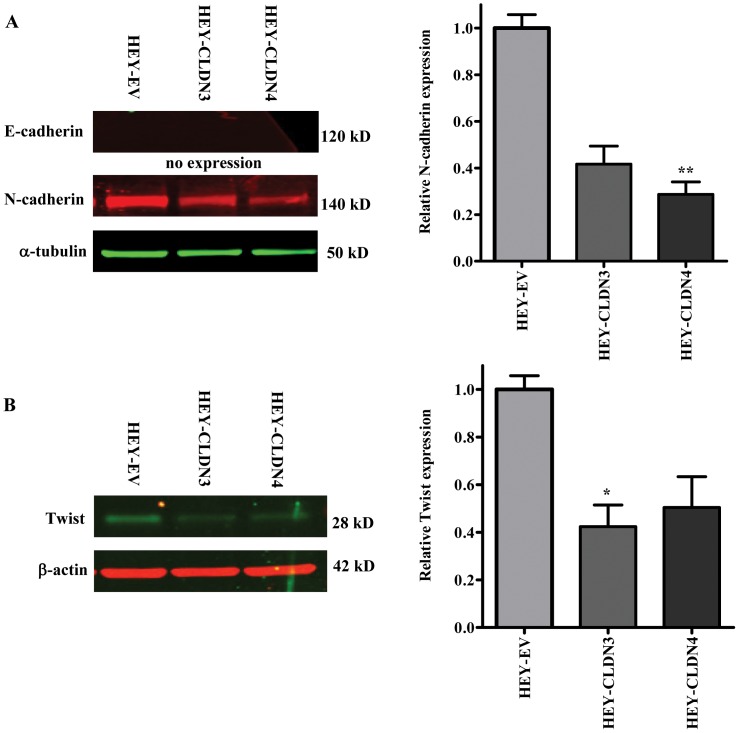
Forced expression of CLDN3 and CLDN4 in HEY cells decreases N-cadherin and Twist expression. Representative Western blot analysis showing reduced expression of N-cadherin (A) and Twist (B) in the HEY cells infected with CLDN3- or CLDN4-expressing lentiviruses. The histograms indicate the levels of the protein determined from 3 independent experiments expressed as the mean ratio relative to that in empty vector control after normalization to α-tubulin or β-actin. Values are mean ± SEM. *p<0.05 *versus* empty vector control; **p<0.01 *versus* empty vector control.

To provide further evidence that the loss of E-cadherin and the gain of N-cadherin and Twist expression occurred as a direct result of the claudin status, we examined the effect of expressing CLDN3 and CLDN4 in the human cancer cell line HEY that does not express endogenous CLDN3 or CLDN4 [34]. HEY cells were infected with lentiviral vectors expressing either CLDN3 or CLDN4 [27], and the constitutively expressing CLDN3 (HEY-CLDN3), CLDN4 (HEY-CLDN4) and empty vector control (HEY-EV) cells were tested for changes in E-cadherin, N-cadherin and Twist levels. While E-cadherin was not detectable in any of the three cell lines, forced expression of either CLDN3 or CLDN4 in the HEY cells down-regulated N-cadherin to 44.5±10.2% or 28.7±5.4% of that in empty vector control (p = 0.094 and 0.001, respectively) (Figure 3A). This is consistent with the observation that CLDN3 or CLDN4 knockdown in the 2008 cells increased N-cadherin expression. Likewise, up-regulation of CLDN3 and CLDN4 also decreased the expression of Twist to 42.4±3.6% and 50.4±13.0% compared to the control (p = 0.024 and 0.062, respectively) (Figure 3B). Thus, both CLDN3 and CLDN4 modulate the expression of two of the most established markers of EMT.

### Re-expression of E-cadherin reverses the effect of CLDN3 and CLDN4 knockdown

**Figure 4:**
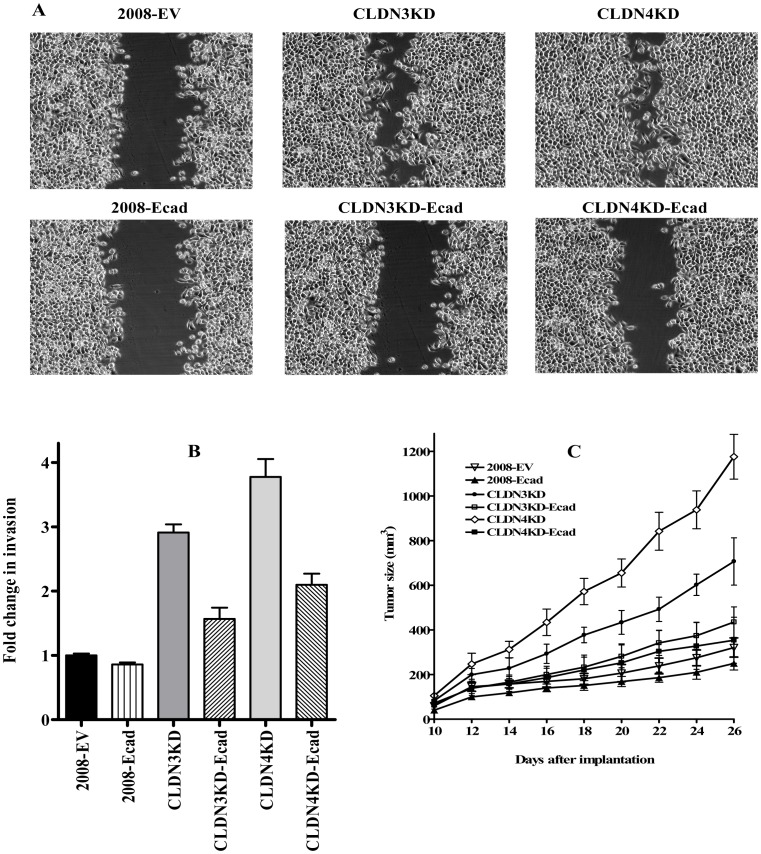
Effect of re-expression of E-cadherin on cell motility, invasion and *in vivo* growth. (A) Relative motility as determined by the ability of 2008-EV, 2008-Ecad, CLDN3KD, CLDN3KD-Ecad, CLDN4KD and CLDN4KD-Ecad cells to close a wound made by creating a scratch through a lawn of confluent cells. Cell images were taken 750 minutes after the scratch. (B) Relative invasion of cells through a layer of Matrigel coated on the filter of a Boyden chamber measured at 24 h after seeding. Bars represent mean ± SEM from 3 independent experiments. (C) Relative growth rate of 2008-EV (∇), 2008-Ecad (▴), CLDN4KD (◊) and CLDN4KD-Ecad (▪) tumors after SC inoculation of 1×10^6^ cells in nu/nu mice (n = 8 for each tumor type). Vertical bars, ± SEM.

To determine whether the observed phenotypic changes characteristic of EMT were attributable to the loss of E-cadherin in the CLDN3KD and CLDN4KD cells, we re-expressed E-cadherin in the CLDN3KD and CLDN4KD cells and compared their migratory and invasive capabilities *in vitro* as well as their growth rate *in vivo*. A wound-healing/scratch assay was used to qualitatively assess cell migration since the extent of wound closure can be taken as a direct measure of cell motility. In agreement with previous findings [27], the CLDN3KD cells closed the wound more rapidly than the parental 2008 cells and the effect was even greater for the CLDN4KD cells. Re-expression of E-cadherin reversed this effect and caused the CLDN3KD and CLDN4KD cells to close the wound more slowly (Figure 4A).

The effect on invasive potential was examined using a modified Boyden chamber invasion assay. The number of cells that invaded through a layer of Matrigel was analyzed at 24 h after plating the cells on Matrigel-coated Transwell inserts. As shown in Figure 4B, knockdown of CLDN3 or CLDN4 in the 2008 cells significantly increased their invasive potential. Infection of CLDN3KD and CLDN4KD cells with a vector expressing E-cadherin substantially reduced cell invasion through the Matrigel while infection with the empty vector had no discernible effect. Forced expression of E-cadherin in the 2008 cells, which already express substantial E-cadherin, had no effect. To determine whether E-cadherin re-expression affected tumor growth *in vivo*, we inoculated 2008-EV, 2008-Ecad, CLDN3KD, CLDN3KD-Ecad, CLDN4KD and CLDN4KD-Ecad cells subcutaneously into nude mice. Similar to the previous observation [27] the CLDN4KD tumors grew much faster than the parental 2008 tumors. However, re-expression of E-cadherin in the CLDN3KD cells, and particularly in the CLDN4KD cells, markedly reduced tumor growth rate whereas there was little effect when E-cadherin was expressed in the parental 2008 cells (Figure 4C). These results suggest that it is the loss of E-cadherin that accompanies down-regulation of CLDN3 or CLDN4 expression that drives the change in migration, invasion and *in vivo* growth rate.

**Figure 5:**
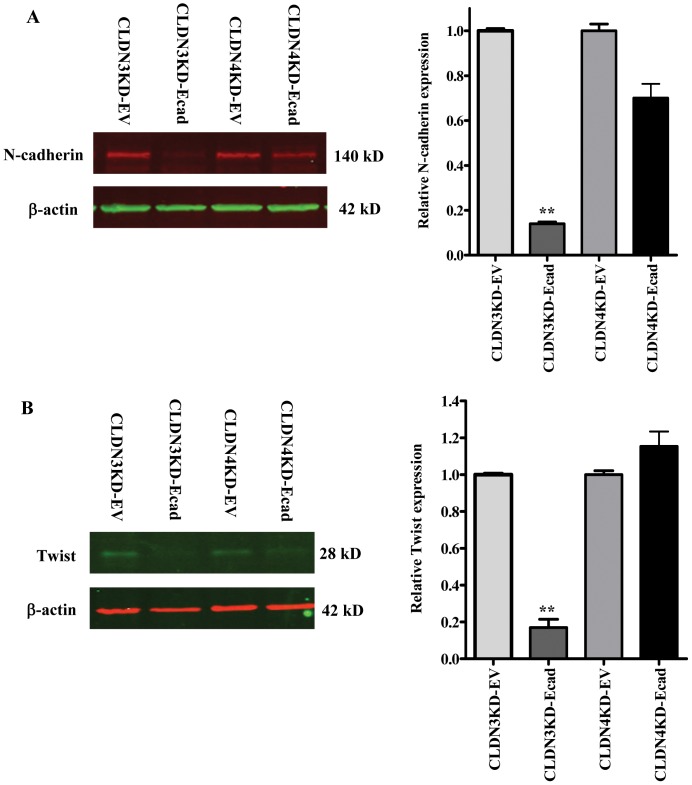
Effect of re-expression of E-cadherin on expression of N-cadherin and Twist. Representative Western blot analysis showing reduced expression of N-cadherin (A) and Twist (B) in the CLDN3KD and CLDN4KD cells stably transfected with pcDNA3.1-E-cadherin-GFP vector. The histograms indicate the levels of the protein determined from 3 independent experiments expressed as the mean ratio relative to that in empty vector control after normalization to β-actin. Values are mean ± SEM, n = 4. **p<0.01 *versus* empty vector control.

To determine whether re-expression of E-cadherin also reversed the effect of CLDN3 and CLDN4 knockdown on the expression of the EMT markers, the levels of N-cadherin and Twist were assessed by Western blot analysis in the E-cadherin re-expressing cells. As shown in Figure 5A, re-expression of E-cadherin in the CLDN3KD cells reduced N-cadherin to 14.0±1.6% of that in the CLDN3KD-EV control cells (p = 0.000014). In the CLDN4KD cells there was a much smaller effect and re-expression of E-cadherin reduced N-cadherin to 69.9±12.7% of that in the CLDN4KD-EV control cells (p = 0.097). Likewise, Twist levels were reduced to 17.0±4.5% (p = 0.0003) in the CLDN3KD-Ecad cells but there was no significant change when E-cadherin was over-expressed in the CLDN4KD cells (Figure 5B). Thus, re-expression of E-cadherin not only reversed the knockdown phenotype, it also reversed the effect of CLDN3 knockdown on the markers of EMT.

### Knockdown of CLDN3 and CLDN4 activates PI3K/Akt signaling

**Figure 6:**
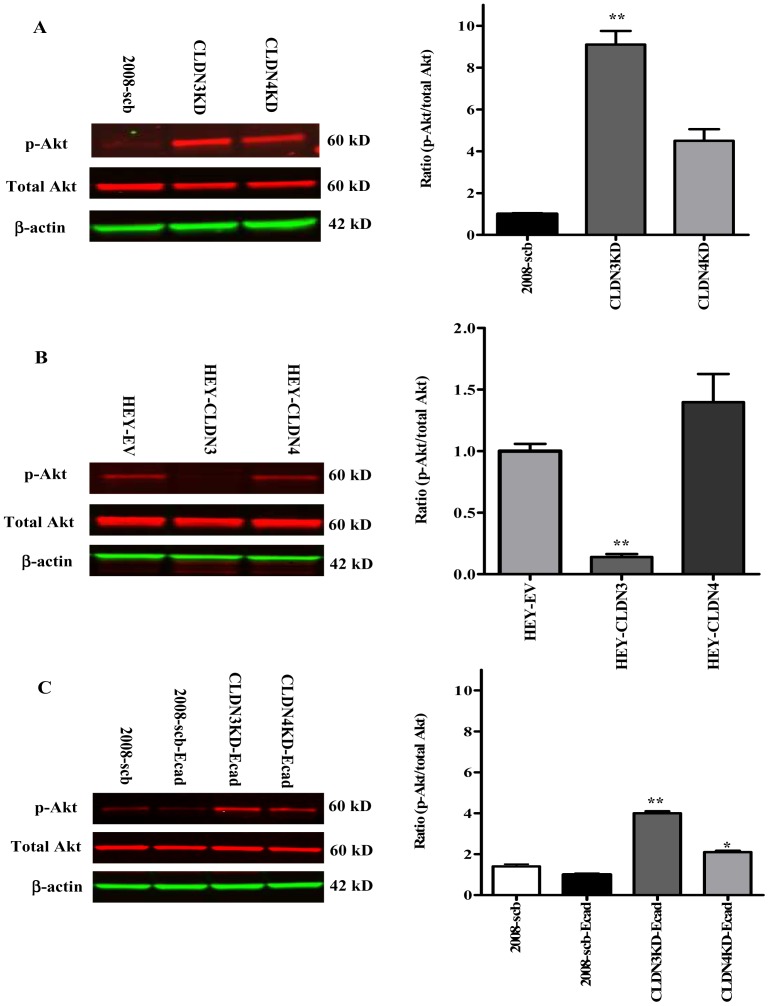
Knockdown of CLDN3 or CLDN4 actives the PI3K/Akt pathway. (A) Knockdown of CLDN3 and CLDN4 in 2008 cells increased Akt phosphorylation. The extent of Akt phosphorylation was measured by Western blot analysis using anti-phospho-Akt antibody and expressed as the ratio relative to that in the scrambled control after normalization to total Akt levels. Values are mean ± SEM, n = 6. **p<0.01 *versus* scrambled-shRNAi control. (B) Ectopic expression of CLDN3 in HEY cells inhibited Akt phosphorylation. Levels of phosphorylated Akt in the HEY-CLDN3 and HEY-CLDN4 cells were expressed as the ratio relative to that in empty vector counterpart after normalization for total Akt. Values are mean ± SEM, n = 3. **p<0.01 *versus* empty vector control. (C) Re-expression of E-cadherin in 2008-scb, CLDN3KD and CLDN4KD cells reduced Akt phosphorylation. Levels of phosphorylated Akt in E-cadherin stably transfected 2008-scb, CLDN3KD and CLDN4KD cells were expressed as the ratio relative to that in 2008-scb-Ecad cells after normalization for total Akt. Values are mean ± SEM, n = 3. *p<0.05 and **p<0.01 *versus* 2008-scb-Ecad control.

The large effect of knocking down CLDN3 and CLDN4 on *in vivo* growth rate suggests a coordinated increase in signaling in multiple pathways. We previously reported that knockdown of CLDN3 or CLDN4 activated β-catenin transcriptional activity [27]. Since activation of the PI3K pathway has been shown to promote EMT in a variety of epithelial cancer cells [37], [38], we assessed the activity of this pathway by quantifying the phosphorylation status of Akt, a direct downstream effector of PI3K. Figure 6A shows a representative Western blot indicating a significant increase in phospho-Akt in the CLDN3KD and CLDN4KD cells by a factor of 9.1±1.6 (p = 0.004) and 4.5±1.4 (p = 0.052) fold, respectively, compared with the 2008-scb cells. Forced expression of CLDN3 in the HEY cells significantly reduced the level of phospho-Akt to 14.0±4.0% of control (p = 0.002) while forced expression of CLDN4 had no effect (Figure 6B). As shown in Figure 6C, re-expression of E-cadherin in the CLDN3KD cells partially suppressed the increased pAkt level that accompanied CLDN3 knockdown, and the same thing was observed when E-cadherin was re-expressed in the CLDN4KD cells. The elevated level in the CLDN3KD cells was reduced by 2.3±0.3-fold (p = 0.036), and that in the CLDN4KD cells by 2.1±0.2 -fold (p = 0.024), when E-cadherin was re-expressed. Forced expression of E-cadherin in the control 2008-scb cells only resulted in 1.4±0.3-fold reduction (p = 0.060) in pAkt. Thus, loss of CLDN3 or CLDN4 expression was associated with large increases in pAKT that were partially offset by rescue of claudin expression or over-expression of E-cadherin.

**Figure 7:**
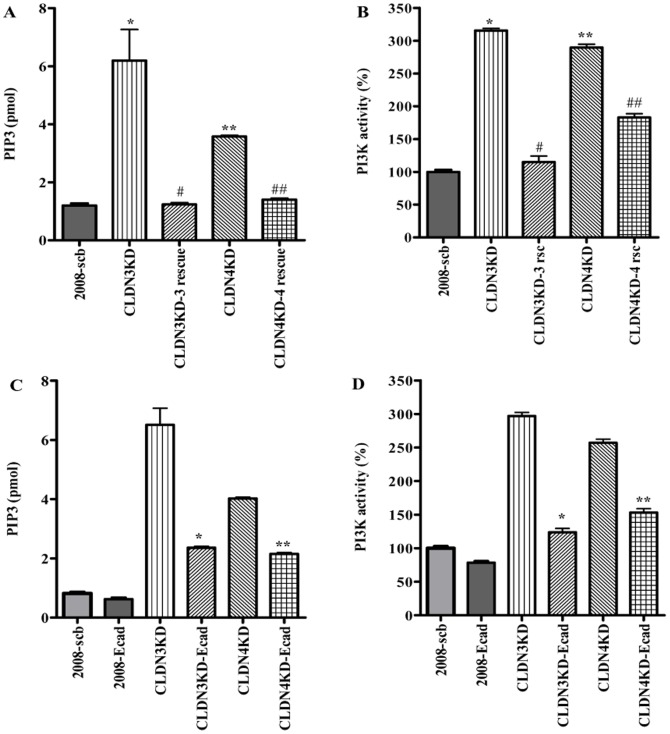
Effect of rescuing expression of CLDN3 and CLDN4, or re-expression of E-cadherin, on PIP3 level and PI3K activity. (A) The cellular content of PIP3 was quantified using a competitive PIP3 ELISA; *p<0.05; **p<0.01 *versus* 2008-scb control; ^#^p<0.05 *versus* CLDN3KD; ^##^p<0.01 *versus* CLDN4KD. (B) Activity of PI3K immunoprecipitated with antibody to p85 regulatory subunit of class IA PI3K; *p<0.01; **p<0.01 *versus* 2008-scb control;^ #^p<0.01 *versus* CLDN3KD; ^##^p<0.01 *versus* CLDN4KD. (C) PIP3 levels; *p<0.001 *versus* CLDN3KD; **p<0.001 *versus* CLDN4KD. (D) Activity of PI3K; *p<0.001 *versus* CLDN3KD; **p<0.001 *versus* CLDN4KD. Values are mean ± SEM, n = 3. PI3K activity is expressed as percent of control.

Akt can be activated by PI3K-dependent or -independent pathways. To determine whether the knockdown of CLDN3 or CLDN4 results in Akt activation as a result of an increase in PI3K activity we measured both the level of PIP3, the product generated by PI3K, and the activity of the immunoprecipitated PI3K itself. As shown in Figure 7A, knockdown of CLDN3 increased the steady-state PIP3 level by 6.2±1.51-fold (p = 0. 043), and knockdown of CLDN4 increased it by 3.58±0.6-fold (p = 0. 001). In both cases re-expression of the respective claudin reversed this effect. Figure 7B shows that the same pattern was observed when the activity of PI3K was measured directly. Knockdown of CLDN3 increased PI3K activity by 3.2±0.3-fold (p = 0.001), and knockdown of CLDN4 increased it by 2.9±0.5-fold (p = 0.001); in both cases re-expression of an shRNAi-resistant claudin reversed this effect. Next, we investigated the effect of re-expressing E-cadherin on PIP3 levels and PI3K activity in the CLDN3KD and CLDN4KD cells. As shown in Figure 7C and 7D, forced expression of E-cadherin in the CLDN3KD and CLDN4KD cells reduced both the elevated PIP3 levels and the PI3K activity. Taken together, these results indicate that CLDN3 and CLDN4 regulate PI3K activity as evidenced by the increased PIP3 level and activation of Akt, and that this is largely mediated through the effect of these claudins on E-cadherin.

## Discussion

The mechanisms that control intercellular adhesion are central to the process of invasion and metastasis. Normal epithelial cells are held together by TJ, adherens junctions and gap junctions. These serve two roles: they mechanically link cells; and, they generate signals that are sent to the interior of the cell to report on the extent of contact with neighbors and the extracellular matrix [39], [40]. One of the hallmarks of malignant transformation of epithelia is that these connections, particularly TJ, are lost [41], [42], [43], [44]. Disassembly or remodeling of TJs can cause loss of cell polarity and an increase in motility and invasiveness [4], [45], [46], and there is an association between the loss of cell-cell adhesion structures and metastasis in many epithelial cancers [47]. The cuboidal layer of cells on the surface of the normal ovary is derived embryologically from mesenchyme. Those tumors that originate in this epithelium appear to undergo a MET to produce the tumors that we know as epithelial ovarian cancers. CLDN3 and CLDN4 are major structural molecules that participate in the formation of TJ between epithelial cells. While normal ovarian surface cells do not express CLDN3 and CLDN4 these two proteins are up-regulated and expressed at high levels in as many as 92% of ovarian cancers presumably as a result of the MET [18], [22], [48]. In fact, CLDN3 and CLDN4 are among the most highly up-regulated genes found in ovarian cancers [17], [18], [21], [49], [50], [51]. However, there is an increasing body of evidence suggesting that serous ovarian cancers arise from the distal Fallopian tubes rather than the ovarian surface epithelium [23], [24], [25], [26]. We have previously performed an immunohistochemical analysis of the expression of CLDN3 and CLDN4 in the Fallopian tubes of 6 patients with serous ovarian cancer and found that both the distal Fallopian tubes and serous ovarian cancers themselves expressed these two proteins in abundance [27]. This new information suggests that serous ovarian cancer may arise in an epithelium that normally expresses CLDN3 and CLDN4 and supports the concept that those cancers that continue to express these proteins are less aggressive than those in which their expression is lost mimicking what is known about “high” versus “low” claudin breast cancers.

Most cells express multiple claudin isoforms that interact in a homotypic and heterotypic fashion to regulate junctional permeability and confer the selectivity and strength to the tight junctions. At least five pairs of claudin genes, including the CLDN3 and CLDN4 pair, have been found to be linked with respect to their similarity in sequence and close proximity in the human genome [52]. This genomic arrangement suggests that gene duplication has played a role in evolution of many of these claudins. We and others have reported that coordinate expression of CLDN3 and CLD4 exists in several normal and neoplastic tissues [27], [53]. Whether the genomic arrangement contributes to coordinate regulation is currently unclear but, at least in the case of CLDN3 and CLDN4, a recent study has identified grainyhead-like 2 (Grhl2) as a transcription factor that is capable of coordinately regulating the expression of both [54].

The concept that the claudins of the TJ can regulate tumor cell behavior has precedence in the well-documented ability of E-cadherin, the major structural protein of the adherens junction, to do this. Loss of E-cadherin is a hallmark of the EMT through which many investigators believe tumor cells must pass to become metastatic [4], [55], [56], [57]. Passage through the EMT is widely reported to result in increased growth rate, enhanced metastatic potential, drug resistance and the acquisition of stem cell characteristics [58]. Although E-cadherin is expressed in the vast majority of ovarian carcinomas there are some subtypes of ovarian cancer such as serous and clear cell carcinomas that have been reported to have weak to absent E-cadherin staining that is associated with cancer progression and more extensive peritoneal dissemination [59], [60], [61]. In agreement with our results, HEY cells that do not express CLDN3 and CLDN4 also do not express E-cadherin, which may explain why the HEY cell line was the most aggressive among 5 ovarian tumor lines tested [62] and indicates that CLDN3 and CLDN4-dependent transcriptional regulation of E-cadherin may already exist in these cells.

The morphological changes that occur during EMT are accompanied by alterations in the expression of a large number of molecules. Epithelial markers that are down-regulated during this process include E-cadherin, cytokeratins, ZO-1, claudins, occludin, laminin-1, entactin, MUC1, and the microRNA 200 (miR-200) family [29]. Molecules that are up-regulated in this process include the transcription factors Snail, Slug, Twist, Zeb1 and Zeb2/SIP1, E47, KLF8, E2.2, Goosecoid, LEF-1 and FoxC2, as well as N-cadherin, vimentin, fibronectin, miR10b, and miR21 [4], [63], [64]. Although many of these molecules can be altered during embryogenesis and cancer-related EMT, tumor cells differ from embryonic cells in having activated signal transduction pathways that promote angiogenesis and suppress apoptosis [29]. In our experimental system, we found that knockdown of either CLDN3 or CLDN4 resulted in down-regulation of E-cadherin and up-regulation of N-cadherin and Twist but did not affect the expression of vimentin, snail, slug and Zeb2. Our observation that decreased expression of E-cadherin in the CLDN3 and CLDN4 knockdown cells was accompanied by an increase in the expression of EMT markers is consistent with the “cadherin switch” whereby a decrease in E-cadherin level results in an increase in N-cadherin expression. Such changes in cadherin expression have been observed in a variety of cancers and experimentally linked to increased cell motility, invasion and metastasis [65]. An important contribution of the loss of E-cadherin to tumor cell aggressiveness is further supported by our finding that restoration of E-cadherin substantially reduced cell migration, invasion *in vitro* and growth rate *in vivo*. The permissive signal that allows progression of the EMT program remains largely unknown. We have previously shown that loss of CLDN3 or CLDN4 led to a large reduction in E-cadherin causing release of β-catenin into the cytosol and inactivation of GSK3β, which facilitated its nuclear translocation to activate LEF/TCF-mediated transcription [27]. However, while β-catenin is necessary for several aspects of the EMT induced by loss of E-cadherin it may not be sufficient to cause these phenotypes [66]. Several studies suggest that β-catenin-mediated transcription can further induce the expression of EMT-related transcription factors such as Twist [66], [67], [68] ultimately contributing to the EMT program. Twist has been demonstrated to bind the E-cadherin promoter via the E-box, as do Snail and Zeb2 [69], [70], to suppress its transcription, thus facilitating EMT and metastatic spreading of epithelial tumors [71], [72], [73]. Our data indicate an inverse association between Twist and E-cadherin and Twist and CLDN3 and CLDN4, further attesting to its role in EMT and tumor progression.

Recently, activation of the PI3K/Akt axis has been identified as a central feature of EMT in tumor cell lines and clinical samples [11], [37], [74]. AKT was initially described as an oncogene involved in many basic cellular processes including cell cycle progression, cell proliferation, cell survival, metabolism and EMT [37]. The Akt-induced EMT is characterized by loss of cell–cell adhesion, morphological changes, loss of apico-basolateral cell polarization, induction of cell motility, decrease in cell–matrix adhesion, and changes in the production or distribution of specific proteins [75]. At the molecular level, Akt activation in epithelial cells results in transcriptional repression of the E-cadherin gene and concentration of a small amount of E-cadherin protein in perinuclear organelles [75], which allows the cell to remain in a mesenchymal state during the exponential growth phase. Although it is not clear whether a defective E-cadherin/catenin complex is the sole factor that determines the invasive phenotype, the ability of forced expression of E-cadherin to reverse the invasive phenotype, the increased PI3K activity and enhanced Akt phosphorylation induced by loss of CLDN3 and CLDN4 indicates that it plays a central role. Taken together, our results suggest that CLDN3 and CLDN4 serve to maintain the expression of E-cadherin which acts as a central modulator of EMT in the ovarian cancer cells via a pathway involving PI3K/Akt and EMT transcription factor Twist. The present observations raise the possibility of exploiting CLDN3 and CLDN4 as potential biomarkers for ovarian cancer progression and highlight maintenance of E-cadherin levels as a target for therapeutic intervention.
